# The Role of 3 Tesla Diffusion-Weighted Imaging in the Differential Diagnosis of Benign versus Malignant Cervical Lymph Nodes in Patients with Head and Neck Squamous Cell Carcinoma

**DOI:** 10.1155/2014/532095

**Published:** 2014-06-09

**Authors:** Flavio Barchetti, Nicola Pranno, Guglielmo Giraldi, Alessandro Sartori, Silvia Gigli, Giovanni Barchetti, Luigi Lo Mele, Luigi Tonino Marsella

**Affiliations:** ^1^Department of Radiological Sciences, Oncology and Pathology, Sapienza University of Rome, 00186 Rome, Italy; ^2^Department of Oral and Maxillofacial Sciences, School of Dentistry, Sapienza University of Rome, 00186 Rome, Italy; ^3^Department of Public Health and Infectious Diseases, Sapienza University of Rome, 00185 Rome, Italy; ^4^Department of Biomedicine and Prevention, Tor Vergata University, 00133 Rome, Italy

## Abstract

*Objective*. The aim of this study was to validate the role of diffusion-weighted imaging (DWI) at 3 Tesla in the differential diagnosis between benign and malignant laterocervical lymph nodes in patients with head and neck squamous cell carcinoma (HNSCC). *Materials and Methods*. Before undergoing surgery, 80 patients, with biopsy proven HNSCC, underwent a magnetic resonance exam. Sensitivity (Se) and specificity (Spe) of conventional criteria and DWI in detecting laterocervical lymph node metastases were calculated. Histological results from neck dissection were used as standard of reference. *Results*. In the 239 histologically proven metastatic lymphadenopathies, the mean apparent diffusion coefficient (ADC) value was 0.903 × 10^−3^ mm^2^/sec. In the 412 pathologically confirmed benign lymph nodes, an average ADC value of 1.650 × 10^−3^ mm^2^/sec was found. For differentiating between benign versus metastatic lymph nodes, DWI showed Se of 97% and Spe of 93%, whereas morphological criteria displayed Se of 61% and Spe of 98%. DWI showed an area under the ROC curve (AUC) of 0.964, while morphological criteria displayed an AUC of 0.715. *Conclusions*. In a DWI negative neck for malignant lymph nodes, the planned dissection could be converted to a wait-and-scan policy, whereas DWI positive neck would support the decision to perform a neck dissection.

## 1. Introduction

The detection of cervical lymph node metastases has a pivotal effect for prognosis and treatment planning of patients with head and neck cancers, as this significantly worsens the treatment outcome. Therefore, there is an increasing need for available noninvasive imaging techniques able to differentiate between benign and malignant lymph nodes [1–8]. According to conventional imaging techniques such as ultrasonography, computed tomography (CT), and conventional anatomical magnetic resonance imaging (MRI) based on T1 and T2 weighted sequences, the parameters used are size, shape, and changing in internal architecture of the lymph nodes. Since differences in T1 and T2 relaxation do not enable reliable nodal differentiation, MRI has not yielded any advantage in addition to CT in the detection of small nodal metastases [9]. As a consequence, conventional MRI, with morphologic criteria for nodal staging limited and similar to those of CT, has generally yielded results that are similar or slightly inferior to those of CT [10]. Molecular imaging based on positron emission tomography (PET) scanners improves the diagnostic accuracy of conventional imaging techniques.

Nowadays there is an increasing interest in diffusion-weighted imaging (DWI), which is an emerging noninvasive functional MRI technique that can be acquired without the administration of intravenous contrast agent and seems to be quite reliable in distinguishing between benign and malignant tissues [11]. DWI provides image contrast dependent on the molecular motion of water, and any architectural changes in the proportion of extracellular to intracellular water protons—like metastases in lymph nodes—will alter the diffusion coefficient of the tissue.

Previous studies performed in a small number of patients with head and neck squamous cell carcinoma (HNSCC) showed that DWI using 1.5 Tesla magnet displayed promising results in differentiating benign from metastatic cervical lymph nodes [12–16].

In this setting, the purpose of our prospective study was to validate the diagnostic accuracy of DWI at 3 Tesla in the differential diagnosis between benign and malignant laterocervical lymph nodes in a larger cohort of patients with HNSCC, using histological results from radical neck dissection as the standard of reference.

## 2. Materials and Methods

### 2.1. Patient Population

This prospective study was approved by the local ethics committee, and all patients gave written informed consent prior to being included. From January 2009 to December 2012, a series of 80 consecutive patients (49 males and 31 females, age range 34–75 years) was enrolled in this study. The patients were included if they had a biopsy proven HNSCC and nonpalpable laterocervical lymphadenopathies and were scheduled for surgery including neck dissection. The patients with palpable laterocervical lymphadenopathies or who had been treated previously for head and neck cancer were excluded from the study. Patients meeting the inclusion criteria were included in the study regardless of clinical tumor stage, nodal stage, or tumor location.

All patients underwent MRI as part of the routine diagnostic workup in order to assess the locoregional staging of the disease.

### 2.2. MRI Technique

All examinations were performed with a 3 Tesla magnet (Discovery MR750, GE Healthcare, Milwaukee, USA) using a dedicated coil for head and neck studies. The range was from the base of skull to the level of the clavicles. To ascertain correlation of the conventional images and DWI, all sequences were acquired with similar geometry.

The conventional MR protocol consisted of axial T2-weighted (TR/TE 1704/80), axial and coronal T1-weighted (TR/TE 400/12), contrast-enhanced axial and coronal fat-suppressed T1-weighted (TR/TE 740/12), and coronal short tau inversion recovery (TR/TE/TI 5868/60/15) sequences. Slice thickness was 3 mm; there was no interslice gap. The matrix was 272 × 512 with a field of view 220 (rectangular field of view (RFOV) 80%).

DW images were acquired in the axial plane by using a gradient single-shot echo planar imaging sequence. The sequence was repeated for two b values, b = 0 and b = 1000 sec/mm^2^, with the following parameters: TR/TE 5666/70, section thickness 3 mm according to the conventional protocol, the matrix was 272 × 512 with a field of view 400 (RFOV 80%), there was no interslice gap, and the number of signal acquisition was six. A total of 64 slices were acquired which covered the whole neck region from the skull base to the clavicles. The total scan time was 4 min and 54 sec. DWI was performed before the contrast enhanced T1-weighted sequences. Apparent Diffusion Coefficient (ADC) maps were automatically reconstructed by means of a standard postprocessing software imager in the main console.

### 2.3. MRI Interpretation

MR images were reviewed independently by 2 radiologists blinded to clinical information: one with 30 years of expertise and the other with 5 years of experience in head and neck imaging. Any disagreement regarding image findings was resolved by consensus.

According to morphological criteria, lymph nodes were assumed to be suspicious for metastatic involvement if they showed one of the following features:

- oval in shape with a maximum transverse diameter greater than 10 mm,
- round shaped and exceeded 8 mm in diameter,
- any size and shape with internal central or eccentric necrotic areas,
- any size and shape with spiculated or indistinct borders and heterogeneous signal intensity (SI) on fat-suppressed T2-weighted images.

As regards DW images interpretation, to ascertain a node-by-node correlation between the conventional images and the DW images, all lymph nodes on the T1-weighted and T2-weighted images were related to the corresponding b1000 images. On these b1000 images, a region of interest (ROI) was drawn manually around each single lymph node, the ROI was copied to the ADC map, and the ADC value was automatically calculated by the postprocessing software of the MR machine. The final whole-node ADC value was obtained by drawing an ROI covering the whole node in all sections in which it was present. The results where averaged and expressed as mean value ± standard deviation. If a necrotic area in a lymphadenopathy determined as a hyperintense region on the T2-weighted image corresponded to a hypointense region on the contrast-enhanced T1-weighted images, it was excluded from the area of analysis.

To reduce the effects of partial volume artifacts, the smallest lymph node size for ADC calculation in a lymph node was set at a minimal axial diameter of 4 mm [16].

### 2.4. Pathological-Radiological Correlation

All neck dissection specimens were removed en bloc by the study's surgeons and divided in the operating room into anatomical neck levels according to American Academy of Otolaryngology/Head and Neck Surgery criteria. The results of the measurements on MRI were compared with the results of the pathological examination of the neck dissection specimens. By recording the combination of the maximum short axial diameter and the exact location of each lymph node per neck level—related to surrounding anatomic structures such as blood vessels, muscles, and salivary glands—it was possible to perform a topographic correlation for each lymph node between the pathological examination and the MR images.

### 2.5. Statistical Analysis and Clinical Validation

Sensitivity (Se), specificity (Spe), positive predictive value (PPV), negative predictive value (NPV), and accuracy of conventional criteria and DWI in detecting laterocervical lymph node metastases were calculated on per-lesion basis analysis. Receiver operating characteristic (ROC) curves were developed in order to compare the diagnostic accuracy between conventional MRI criteria and DWI results. Histological results with respect to presence or absence of lymph node metastases were used as standard of reference.

The data were evaluated using the statistical analysis software SPSS 19.0 for Windows (SPSS, Statistical Package for Social Science, IBM Corporation, Armonk, NY, USA). In all analyses, *P* values < 0.05 were considered as indicator for statistical significance.

## 3. Results

A total of 103 neck dissections were performed: 41 supraomohyoid neck dissections, 57 radical neck dissections, and 5 selective neck dissections.

**Figure 1:**
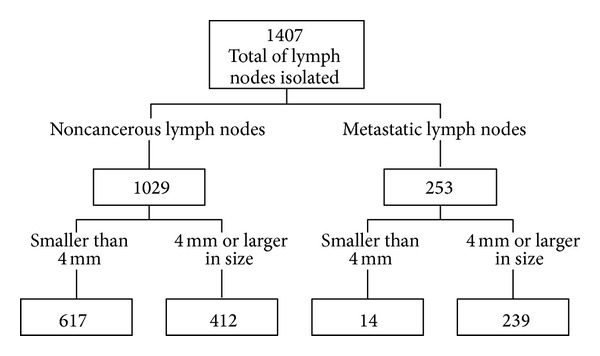
Flow diagram depicting the histopathological findings.

A total of 1407 lymph nodes, predominantly smaller than 10 mm (94.6%), were isolated. At pathological analysis, a total of 253 metastatic lymph nodes were identified: 239 were 4 mm in size or larger and 14 were smaller than 4 mm. 412 out of the 1029 histologically proven noncancerous lymph nodes were 4 mm in size or larger. All the 651 nodes 4 mm or larger in size were identified at DWI (Figure 1).

**Figure 2:**
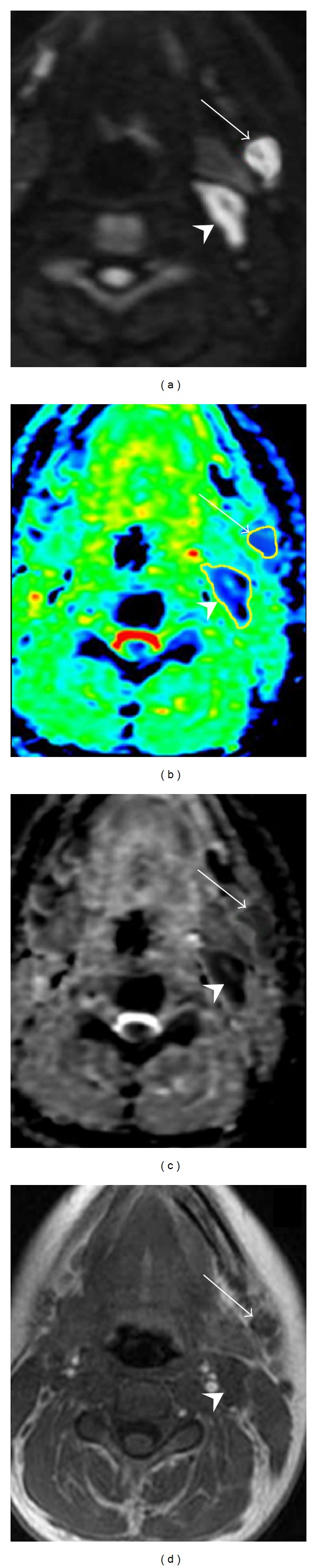
Axial native DWI image at b value of 1000 sec/mm^2^ (a), color ADC map (b), grey scale ADC map (c), and T1-weighted image (d) of a patient with nasopharynx carcinoma showing two solid lymphadenopathies located at level Ib (arrow) and level IIa (head arrow), respectively. According to morphological criteria the lymph node at level Ib, round shaped and 6 mm in size, was considered to be negative for cancer, while the adenopathy localized at level IIa, oval shaped and with a short transverse diameter of 11 mm, was reported to be suspicious for metastatic involvement. Concerning DWI, level Ib lymph node showed a mean ADC value of 0.690 × 10^−3^ mm^2^/sec and was considered suspicious for metastatic involvement, whereas level IIa adenopathy displayed an average ADC value of 1.051 × 10^−3^ mm^2^/sec and, therefore, was deemed to be a noncancerous lymphadenopathy. At pathological examination level Ib lymph node showed large intranodal metastatic deposits and level IIa adenopathy was found to be benign.

**Figure 3:**
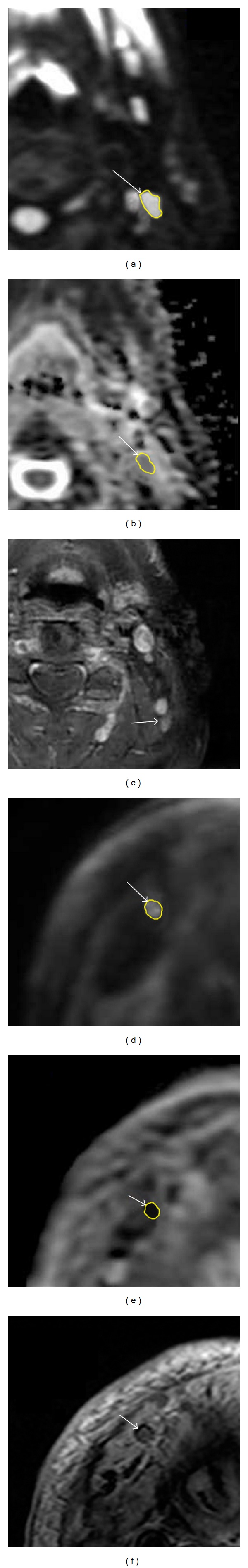
MR images of a patient with a biopsy proven oropharyngeal squamous cell carcinoma. (a) Axial native DWI image at b value of 1000 sec/mm^2^, (b) grey scale ADC map, and (c) contrast-enhanced axial fat-suppressed T1-weighted image showing a left-sided lymph node at level IIa. According to morphological criteria the lymph node oval in shape with a maximum transverse diameter of 6 mm was considered to be noncancerous. At DWI the lymph node showed a mean ADC value of 0.861 × 10^−3^ mm^2^/sec and was considered to be pathologically involved. (d) Axial native DWI image at b value of 1000 sec/mm^2^, (e) grey scale ADC map, and (f) contrast-enhanced axial T2-weighted image showing a right-sided lymph node at level Ib. On the basis of morphological criteria the lymph node, round shaped and 9 mm in size, was considered to be suspicious for metastatic involvement. Concerning DWI, the lymph node showed a mean ADC value of 1.213 × 10^−3^ mm^2^/sec and was deemed to be a benign lymph node. At pathological examination level IIa lymph node showed intranodal metastatic deposits and level Ib lymph node was found to be benign.

**Figure 4:**
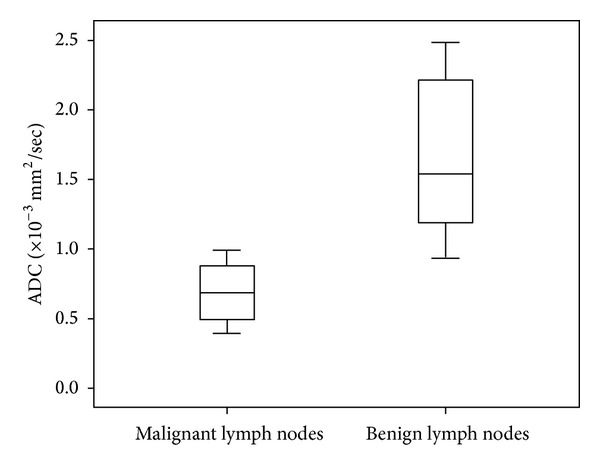
Graph of box plots shows the apparent diffusion coefficient (ADC) values of malignant and benign lymph nodes. The horizontal line in the box represents the median (50th percentile), whereas the top and the bottom represent the 25th and 75th percentile, respectively. The whiskers represent the range from the largest to the smallest measured ADC data. The ADC values of benign lymph nodes are higher compared to those of malignant lymph nodes.

In the 239 histologically proven metastatic lymphadenopathies, the mean ADC value was 0.903 × 10^−3^ mm^2^/sec (range: 0.400–0.996 mm^2^/sec). In the 412 histologically confirmed benign lymph nodes, ADC maps showed an average value of 1.650 × 10^−3^ mm^2^/sec (range: 0.945–2.370 × 10^−3^ mm^2^/sec) (Figures 2, 3, and 4).

The most reliable threshold of ADC values, derived from receiver operating characteristic analysis, for differentiating benign versus metastatic lymph nodes, was 0.965 × 10^−3^ mm^2^/sec, showing a Se of 97%, a Spe of 93%, accuracy of 92%, PPV of 95%, and NPV of 96%.

Morphological criteria showed a Se of 61%, a Spe of 98%, accuracy of 72%, PPV of 65%, and NPV of 87%.

DWI showed an area under the ROC curve (AUC) of 0.964 (standard error (SE) 0.020; 95% confidence interval (CI) 0.926–0.983), whereas morphological criteria displayed an AUC of 0.715 (SE 0.064; 95% CI 0.547–0.818).

**Figure 5:**
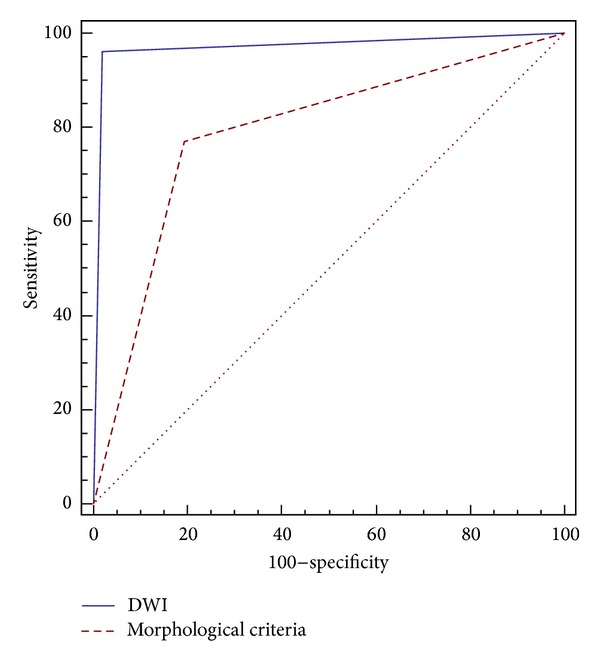
Receiver operating characteristic (ROC) curve comparison for DWI and morphological criteria in the differentiation between benign and metastatic lymphadenopathies.

The pairwise comparison of ROC curve values was 0.249 (SE 0.057; Z statistic 5.019; CI 95% 0.173–0.339; *P* = 0.005) (Figure 5).

## 4. Discussion

Squamous cell carcinoma (SCC) is one of the most frequent tumours in the head and neck region for which chemoradiotherapy (CRT) and surgery are curative treatment options. Metastatic involvement of neck lymph nodes is a frequent finding for this tumour type and has a major negative prognostic impact on patient survival [17]. For this reason, accurate detection of nodal metastases is mandatory to optimize the treatment plan. It is well known that palpation is an inaccurate technique to stage cancer in the neck, with a high percentage of false-negative and false positive findings. Because of this inaccuracy, a policy of prophylactic neck treatment, with either radiation therapy or surgery, is widely accepted if the risk of occult metastases is estimated to be above 15%–20% [18–20]. This risk of occult metastases, which can occur in both sides of the neck, is determined by characteristics of the primary tumour such as size, site, and several biological criteria. Basically, most patients with tumours staged as T2 or larger undergo some form of elective neck treatment. Although the most important advantage of elective treatment is that the policy warrants an as early as possible therapy for occult metastases, the disadvantages are overtreatment and the high morbidity rate.

Theoretically, neck nodes need no treatment if no metastases are present. Accurate pretreatment staging of disease with modern imaging techniques can provide substantial evidence on the status of the neck nodes. If the neck remains N0 after an accurate staging procedure, the risk of occult metastases might be diminished below 10–15% depending on the initial risk of occult metastases, that is, the size and the site of primary tumour. Such a low risk of occult metastases can be a reason to refrain from elective treatment and just provide careful follow-up of the patient [19]. In cases in which lymph node metastases occur during follow-up, a delayed therapeutic neck dissection can be performed. To minimize the treatment delay, such a recurrence should be detected as early as possible (preferably before it becomes palpable) with use of an accurate imaging technique. For patients in whom cancer is upstaged from NO to N+ with the use of accurate imaging techniques, the planned wait-and-see policy can, in an early phase, be changed to a therapeutic procedure.

CT and MRI are the primary diagnostic modalities for locoregional staging of HNSCC. However, their reliance on morphological and size-related criteria bears some inherent disadvantages. Both techniques may fail to detect small metastatic deposits and have difficulty in differentiating between metastatic and inflammatory enlarged lymph nodes, a problem that is not always solved by [18F]-fluorodeoxyglucose- (FDG-) PET. Moreover, FDG-PET has recognized spatial resolution limitations and cannot reliably identify disease of < 0.5 cm in diameter [21–27]. Although FDG PET/CT has higher sensitivity for the detection of nonpalpable nodal metastases compared with anatomic imaging modalities, its added clinical value in the preoperative assessment of HNSCC has not been proven unambiguously [28, 29]. The low spatial resolution of this technique may lead to false-negative results in cases of small-volume nodal disease or microscopic metastatic invasion in lymph nodes smaller than 1 cm in size.

Therefore, although most radiologists emphasize that these techniques are very accurate, most clinicians have maintained that none of these techniques is perfect and thus refrain from adjusting their management policy in individual cases according to the outcome of these imaging modalities.

Currently, there are compelling clinical and pharmaceutical needs to move towards an ideal diagnostic imaging technique able to assess the cancer burden avoiding radiation exposure and to fulfill some essential prerequisites: accuracy, availability, reproducibility, cost effectiveness, and efficiency [30]. DW-MRI seems to fulfill these requirements, because no ionizing radiation is administered and no intravenous injection of isotopes or any contrast medium is necessary.

DWI is based on an echo-planar sequence and depicts the diffusivity of water molecules along the three space directions within the tissue. It provides qualitative and quantitative information reflecting tissue cellularity and cell membrane integrity and therefore complements morphologic information obtained with conventional MRI. The motion of water molecules in extra- and intracellular spaces contributes to the net water displacement measured by DWI. The degree of water molecules diffusion in biologic tissue is inversely correlated to the tissue cellularity and the integrity of cell membranes [31]. Generally, the motion of water molecules is more restricted in tissues with a high cellular density and intact cell membranes (e.g., tumour tissue). Qualitative (visual) assessment of relative tissue signal attenuation at DWI is used for tumour detection and tumour characterization, while quantitative analysis of DWI is achieved by calculation of ADC. The ADC is calculated for each pixel of the image and is displayed as a parametric map (ADC map) [32].

The differential diagnosis between benign and malignant lymph nodes is highly dependent on quantitative analysis with ADC calculation. Both benign inflamed and malignant lymph nodes may show an increased but variable SI on DWI native images and low SI on ADC map. Quantitative analysis can be performed by drawing ROIs over the lymph nodes on the separate native b-value images and calculating the ADC from the SI on the consecutive b-value images [33]. In general, solid adenopathies are evaluated placing an ROI over the entire volume of the lymph node. In necrotic adenopathies, ROIs are drawn on the solid portions [34].

The use of 3 Tesla magnet could have in this setting several advantages compared with the standard magnetic field of 1.5 Tesla. Theoretically, the signal-to-noise ratio (SNR) increased twofold on moving from 1.5 to 3 Tesla, and an increased SNR can be translated into improvements in spatial, temporal, and spectral resolution. A limited SNR at 1.5 Tesla may impair MR sensitivity for subtle changes in ADC values. The increase in SNR from 3 Tesla imaging enables consequently an increase in the SNR of the ADC maps, so a possible increase in the accuracy of the measurements of ADC values using ROIs may be expected. Therefore, the potential measurement error for tumor ADC values at 3 Tesla might be lower than that at 1.5 Tesla [35].

Metastatic lymph nodes have significantly lower ADC values than benign lymph nodes [30]. The decreased ADC values most likely correlate to the tumour microstructure, which demonstrates a large number of cells, cellular polymorphism, and increased mitosis. These features probably diminish the extracellular extravascular space and decrease the ADC values [36]. This is in contrast with the normal nodal architecture, which consists mainly of small lymphoid cells ordered in scattered germinal centers and vessel-like sinusoids. These features can be expected to correlate with an enlarged interstitial space, facilitation of water movement, and high ADC values. Nevertheless in necrotic metastases, the low amount of intact tumoural cells can result in increased ADC values, potentially leading to misdiagnosis. The equally low ADC values seen in partially invaded lymph nodes suggest the presence of additional mechanisms. For instance, keratinization and peritumoural nodal reactivity may have contributed to the restricted diffusion [37, 38]. From data in the literature, it is known that keratin impairs water movement; therefore, the presence of keratin—which is specific for SCC metastases—may intensify the ADC decrease in metastatic lymph nodes [37].

False positive findings on DWI are mainly caused by nodal reactivity, which is characterized by a homogenous lymphoid infiltration, organized in a multitude of germinal centers and the presence of fibrous stroma [39]. These histological features also increase microstructural barriers and can decrease ADC values to a level similar to that of metastatic lymph nodes. Careful correlation with nodal morphology may be helpful in avoiding misdiagnosis as the presence of a nodal fat-containing hilus is highly indicative for the absence of metastatic disease [40].

The use of DWI to characterize head and neck lesions and discriminate malignant from benign lymph nodes with higher sensitivity and specificity than anatomical imaging modalities has been investigated in only a limited number of studies [12–16]. DWI has been shown to enable differentiation between malignant and benign lymph nodes with sensitivities ranging from 52% to 98% and specificities ranging from 88% to 97%—albeit with different ADC thresholds [12, 14]. Remarkably and contrary to findings of other studies, Sumi et al. [14] measured higher ADC values for metastatic lymph nodes than for benign lymph nodes. These differences cannot be attributed solely to the different b value settings used for ADC calculations. The high number of necrotic metastatic lymph nodes included in the study of Sumi et al. probably contributed to the discrepancy. On the other hand, in these studies, imaging has been used to characterize enlarged lymph nodes only; however, the clinical endpoint is the identification of metastases in subcentimeter lymph nodes. de Bondt et al. analysed a series of 219 lymph nodes, predominantly smaller than 10 mm (95.4%), from a cohort of 16 patients and, using an optimal ADC threshold of 1.0 × 10^−3^ mm^2^/sec as cutoff point, found Se 92.3%, Spe 83.9%, 43.6% PPV, and 98.8% NPV for differentiating malignant and benign lymph nodes, using neck dissection as gold standard [15].

Vandecaveye et al., using neck dissection as standard of reference, performed a correlation of histopathologic and radiologic findings in 301 lymph nodes from a series of 33 patients. They showed that DWI with use of ADC b0–1000 values had higher accuracy in the detection of metastatic lymph nodes than turbo spin echo (TSE) MRI, mainly by improving the detection of subcentimeter nodal metastases. The use of this technique also improved the identification of benign enlarged lymph nodes. For differentiation of malignant versus benign lymph nodes greater than 10 mm in size, with an optimal ADC b0–1000 threshold of 0.94 × 10^−3^ mm^2^/sec, 94% Se, 80% Spe, 90% accuracy, 94% PPV, and 80% NPV were achieved, whereas 97% Se, 10% Spe, 76% accuracy, 78% PPV, and 50% NPV were found for TSE MRI. To distinguish malignant versus benign lymph nodes between 4 and 9 mm in size, with the same optimal ADC b0–1000 threshold of 0.94 × 10^−3^ mm^2^/sec, 76% Se, 94% Spe, 92% accuracy, 73% PPV, and 95% NPV were achieved, while 7% Se, 99.5% Spe, 85% accuracy, 75% PPV, and 85% NPV were found for morphological criteria. Compared with TSE, DWI had higher Se (76% versus 7%) but slightly lower Spe (94.0% versus 99.5%) for detection of subcentimeter nodal metastases. In the detection of subcentimeter lesions, DWI showed a Se of 76% and a Spe of 94% for the detection of 4–9 mm lymph nodes, while the Se of conventional imaging was 7% for the detection of these lesions [16]. Several factors enable the detection of small nodal metastases at DWI. Use of improved echo-planar imaging technology, dedicated coils, and dedicated sequence optimization enables a maximal reduction of echo-planar imaging-related artifacts at a relatively high spatial resolution [41]. The reduction of these artifacts, in combination with the high gradient strength available with current MRI systems, substantially increases the SNR and thereby propagates the generation of a sufficient signal in small lymph nodes and reduces the influence of noise in ADC calculations. Applying a larger number of b values is likely to reduce both the influence of noise propagation in ADC calculations and the risk of motion-related artifact generation [16].

The results of our study, obtained in a large number of lymph nodes predominantly smaller than 10 mm in size, seem to validate the diagnostic accuracy of DWI at 3 Tesla in the differential diagnosis between benign and malignant cervical lymph nodes in patients with HNSCC. Using a cutoff ADC value of 0.965 × 10^−3^ mm^2^/sec, we reached a Se of 97%, a Spe of 93%, and accuracy of 92%, definitely higher than morphological criteria (Se of 61%, Spe of 98%, and accuracy of 72%).

The main limitation of our study is that not all patients underwent bilateral neck dissection. Therefore, a number of micrometastases in the contralateral neck may have remained undetected. Furthermore, we excluded lymph nodes smaller than 4 mm from analysis because such small nodes would not have permitted adequate ADC quantification.

In conclusion, DWI, due to its ability to probe the tumoural microstructure, its short acquisition time, its high repeatability, and safety given the absence of intravenous administration of contrast medium, could be assumed to be the most noninvasive tool to differentiate between benign and metastatic lymph nodes in patients with HNSCC. DWI provides complementary information to conventional MRI since size-related and morphologic criteria lack sufficient reliability for identification of small nodal metastases in patients with HNSCC. Therefore, in a DWI negative neck, the planned neck dissection could be converted to a wait-and-scan policy, whereas DWI positive neck would support the decision to perform a neck dissection. Moreover a future potential application, which is currently under research, is to use DWI as a predictive biological marker during and early after CRT for head and neck cancer. Unlike FDG-PET, one of the major advantages of DWI is the insensitivity to inflammatory changes, making the technique suitable for tumour imaging during CRT. The increasing in ADC values depicted by DWI may positively correlate with treatment response.

Nevertheless, further studies in which the described promising results are reproduced on a wider series of patients and that are focused on standardization of the imaging technique and the image interpretation are required before DWI can be used in the routine clinical decision making of patients with HNSCC.
